# Tides in the Last Interglacial: insights from notch geometry and palaeo tidal models in Bonaire, Netherland Antilles

**DOI:** 10.1038/s41598-017-16285-6

**Published:** 2017-11-24

**Authors:** Thomas Lorscheid, Thomas Felis, Paolo Stocchi, J. Christina Obert, Denis Scholz, Alessio Rovere

**Affiliations:** 1 MARUM - Center for Marine Environmental Sciences, University of Bremen, Leobener Straße 8, 28359 Bremen, Germany; 2 ZMT - Leibniz Centre for Tropical Marine Research, Fahrenheitstraße 6, 28359 Bremen, Germany; 3 NIOZ - Royal Netherlands Institute for Sea Research, Department of Coastal Systems, and Utrecht University, P.O. Box 59, 1790 AB, Den Burg, Texel, The Netherlands; 4 Institute for Geosciences, Johannes Gutenberg-University Mainz, J.-J.-Becher-Weg 21, 55128 Mainz, Germany; 5 Biogeochemistry and Climate Geochemistry Departments, Max Planck Institute for Chemistry, P. O. Box 3060, 55020 Mainz, Germany; 6 Lamont-Doherty Earth Observatory, Columbia University, 61 Route 9W, Palisades, NY 10964 United States

## Abstract

The study of past sea levels relies largely on the interpretation of sea-level indicators. Palaeo tidal notches are considered as one of the most precise sea-level indicators as their formation is closely tied to the local tidal range. We present geometric measurements of modern and palaeo (Marine Isotope Stage (MIS) 5e) tidal notches on Bonaire (southern Caribbean Sea) and results from two tidal simulations, using the present-day bathymetry and a palaeo-bathymetry. We use these two tools to investigate changes in the tidal range since MIS 5e. Our models show that the tidal range changes most significantly in shallow areas, whereas both, notch geometry and models results, suggest that steeper continental shelves, such as the ones bordering the island of Bonaire, are less affected to changes in tidal range in conditions of MIS 5e sea levels. We use our data and results to discuss the importance of considering changes in tidal range while reconstructing MIS 5e sea level histories, and we remark that it is possible to use hydrodynamic modelling and notch geometry as first-order proxies to assess whether, in a particular area, tidal range might have been different in MIS 5e with respect to today.

## Introduction

Fossil landforms, deposits or bioconstructions can be used as indicators of the relative sea-level (RSL) position during past warm periods^1^, under the assumption that the environment at the time of formation is known and its indicative meaning is quantifiable^2–4^. Once a RSL indicator has been measured in the field, its position with respect to the palaeo RSL needs to be quantified, ideally through comparison with analogue modern environments^3,4^. Only after this quantification, and after the correction for tectonics, glacio-isostatic or other post-depositional effects^5–7^, the elevation of the RSL indicator can be transformed into the local height of palaeo eustatic sea level, that is an essential information to assess past ice volumes and to constrain future ice-sheet and sea-level dynamics^8–10^. Except for the Holocene^11^, the only period of Earth’s history for which a large number of RSL indicators was reported globally is the Last Interglacial and in particular, the Marine Isotope Stage (MIS) 5e^12^.

MIS 5e RSL indicators can be divided into 10 general geomorphological types^3^: among them tidal notches are those that can be most tightly related to modern tidal datums. Tidal notches are undercuttings or indentations that are carved near sea level on limestone coasts^13^. In general, the formation of a tidal notch is related to four main processes: bioerosion, wetting and drying tidal cycles, hyperkarst and mechanic abrasion^14^. All these processes happen at or near sea level: the bioerosion affects mainly the lower, submerged part of the notch^15^, whereas the wetting and drying cycles as well as hyperkarst both have a stronger influence on the upper, subaerial part^14,16^. Mechanic abrasion acts where air and water alternate. Despite an ongoing debate^14,15,17,18^ regarding the relative importance of these processes in shaping tidal notches, several studies^3,14,16,19^ report that the width of a tidal notch (i.e., the vertical distance between the base and the roof) is correlated to the amplitude of the mean tidal range (Great Diurnal range, GT^20^, defined as the distance between the Mean Lower Low Water, MLLW, and the Mean Higher High Water, MHHW)^14^. In contrast, the depth of a tidal notch (i.e., how deep the notch is carved into the cliff) is correlated to the rate, duration and intensity of biological and mechanical erosion^16,21^.

In general, the wider a tidal notch, the greater is the GT at the location where the notch is carved: Antonioli *et al*.^14^ used geometric measurements from 73 modern tidal notches in the Mediterranean Sea to show that, in *‘sheltered areas, the notch width is ~0.3–3.2 times the tidal range, a ratio that seems maintained in exposed sites, although with larger variability’*. As the width of a notch is related to the tidal range of the location where it forms, the comparison of modern and palaeo tidal notches may give the opportunity to go beyond the simple reconstruction of palaeo RSL. In fact, under the assumption that the amplitude of a notch was regulated in the past by the same processes as today in equal ratios, different modern and palaeo notch amplitudes can be correlated to changes in tidal range. Therefore, the comparison of the geometrical properties of a modern and a MIS 5e notch can give a first estimate on possible changes in the tidal range between today and MIS 5e.

Another possibility to quantify palaeo tidal ranges is the use of hydrodynamic models that simulate tidal water-level change. Differences in tidal range over long time periods are, at least for the Quaternary, mostly related to changes in the topography of a coastal area under different sea-level conditions^20^. Thus, it is necessary to reconstruct or estimate a palaeo bathymetry to use as model input. Changes in tidal range following relative changes in sea level (also including Glacial Isostatic Adjustment, GIA^22^) are often taken into account when reconstructing Holocene sea-level histories^23–26^, but they have been considered very rarely on Pleistocene time scales^27,28^.

In this study, we take advantage of the geological record preserved on the island of Bonaire (Leeward Antilles) in the southern Caribbean Sea, where a palaeo and modern tidal notch are preserved at the same location and in the same geographic setting, to investigate the potential of using tidal notches or tidal modelling to reconstruct MIS 5e tidal ranges. We first present the results of a survey of palaeo and modern tidal notches in this area, aiming at establishing their geometry and elevation. Then, we show the results from two simulations of a hydrodynamic model forced with modern satellite-derived tidal constituents^29,30^ and two different input bathymetries. We use our field data and model results to discuss the importance and implications of estimating past changes in tidal range.

## Study Area

The island of Bonaire (Leeward Antilles) is situated ca. 100 km north of the Venezuelan coast, in the southern part of the Caribbean Sea. From a tectonic standpoint, the island is part of the Leeward Antilles Ridge and located between the Caribbean and the South American plates. This setting led to a range of compressional structures since the Pliocene and a general SE-directed tilting of the island^31^. The mild uplift rate of some parts of the island is described at 0.08 mm/a, whereas other parts are not considered to have been uplifted in the Quaternary^32,33^.

**Figure 1:**
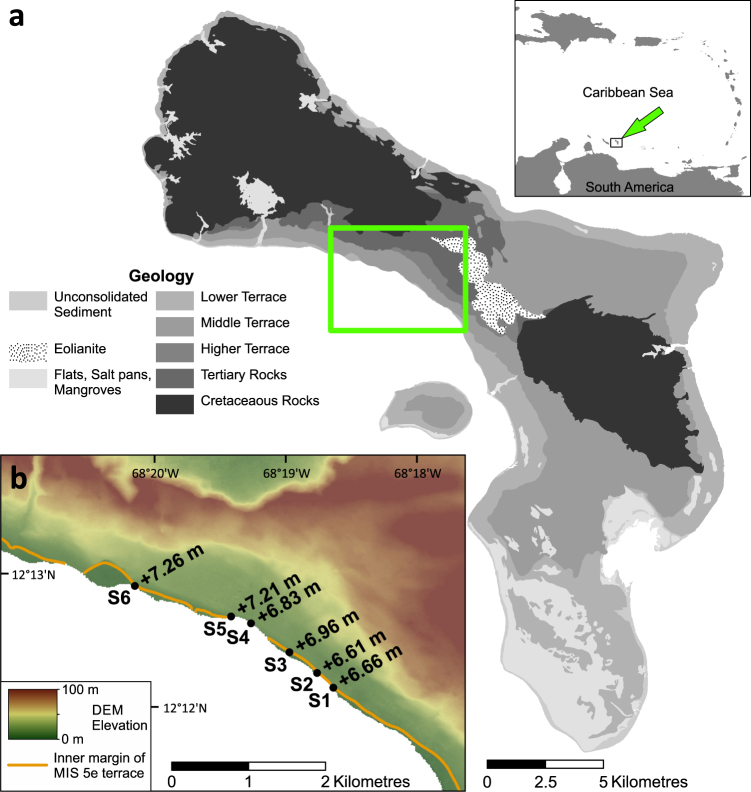
Geology of Bonaire and location of study sites. (**a**) Geological map of the island of Bonaire (modified from Koomen *et al*.^36^). (**b**) Location of the sites, where modern and palaeo notches were measured (S1–S6). Elevations indicate the base of the palaeo notch (red point in Fig. 2a,b). The background map represents the high-resolution Digital Elevation Model (DEM, using the TanDEM-X missions) for this area. The orange line indicates the inner margin of the MIS 5e terrace shown in the cross-section of Fig. 2a (The maps have been created with the software ESRI ArcMap 10.4.1 [http://www.arcgis.com], using data from the DCBD online database [http://www.dcbd.nl/document/geological-map-bonaire] and the TanDEM-X missions). This figure is not covered by the CC-BY licence. TanDEM-X data used in panel b is under copyright by the German Aerospace Center (DLR). All rights reserved, used with permission.

The southern part of the island is dominated by a very flat topography, whereas the north-western and central parts of the island are dominated by a higher topography and large fossil reef terraces (Fig. 1a). The basement of the island is represented by the higher mountains in the North, that consists of Cretaceous-Eocene volcanites, conglomerates and intercalated limestones, followed by Mio-Pliocene limestones^31,34^. On these rocks, four levels of Pleistocene reef terraces developed during different interglacials^35,36^. While these terraces are very wide at the northern and eastern (windward) coastlines (up to 4 km, Fig. 1a), the leeward coast is steeper, and the terraces are limited to a few hundred to tens of meters in width. The lowest of the Bonaire Pleistocene reef terraces has been speculated to have formed during MIS 5e based on chronostratigraphic correlations^35^. This was recently confirmed by strictly reliable U-series ages obtained from corals sampled on the north-eastern and eastern (windward) sides of the island^37–39^. At the southern coastline of the leeward part of the island, steep limestone areas facilitated the formation and preservation of pronounced tidal notches.

**Figure 2:**
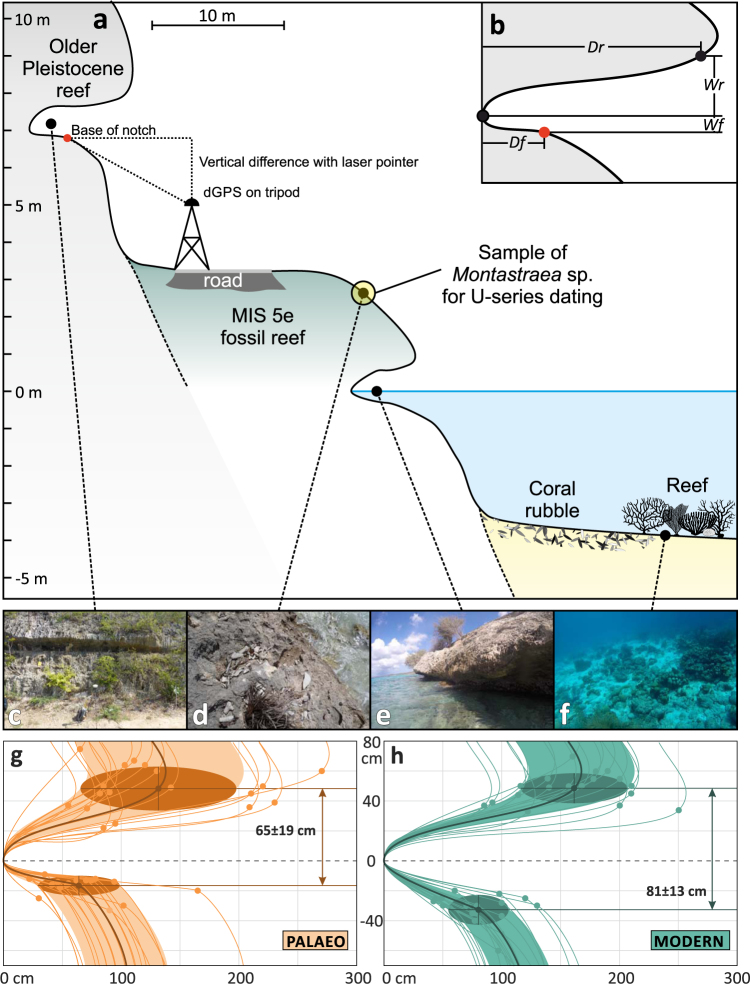
Description of the geometric measurements and field observations. (**a**) Cross-section representing the general morphology of the coastline and the shallow-water reef in the “Tolo” area; (**b**) geometric measures of the notch: *Wr* – upper notch width, *Wf* – lower notch width, *Df* – notch depth at foot, *Dr* – notch depth at roof. Reported dGPS measurements represent the red point (base of notch); (**c**) palaeo tidal notch (section S1 in Fig. 1b); (**d**) MIS 5e fossil reef, from which the *Montastraea* sp. coral has been sampled; (**e**) modern tidal notch (section S3 in Fig. 1b); (**f**) shallow-water reef in the “Tolo” area (−3 to −4 m below sea level). (**g**,**h**) Geometry of palaeo (**g**) and modern (**h**) tidal notches. Dots represent the geometrical nodes represented in b, the bold line and ellipsoid show the mean value and the standard deviations, respectively, for all the measurements. Each line represents one notch measurement (see Methods).

The target of this study is an area locally known as ‘Tolo’, located along the Queen’s Highway, on the leeward side of Bonaire (Fig. 1a,b). The area is characterized by a relatively steep and narrow coastline interrupted by a small fossil reef terrace (5–10 m wide), which forms the lowest fossil reef terrace along this part of the coast. About 2–3 m above this terrace, a palaeo tidal notch is carved into older Pleistocene limestone (Fig. 2a). A modern tidal notch can be observed at sea level (Fig. 2a), and both modern and palaeo notches can be traced laterally for ca. 4 km (Fig. 1b). We measured both the modern and the palaeo notch at six sites, replicating our measurements three times per site (see Methods for details).

## Results

### Modern and palaeo tidal notches

Both the modern and palaeo tidal notches along the north-western coast of Bonaire have a similar geometry, with an overhanging roof and a relatively narrow floor (Fig. 2g,h). The geometry of both tidal notches shows some variability within the different sites (Supplementary Table S1). We derived general geometric properties for modern and palaeo tidal notches by averaging all 18 measured notch profiles. The average width of the palaeo tidal notch is 65 ± 19 cm (Fig. 2g), while that of the modern one is 81 ± 13 cm (Fig. 2h). Overall, the difference in width (i.e., the amplitude of the notch, *Wr* + *Wf* in Fig. 2b) between the modern and the palaeo tidal notch is 16 ± 26 cm, and therefore insignificant within error. The depth of the notch (i.e., how deep into the rock the notch is carved, *Df* and *Dr* in Fig. 2b) is on average larger in the modern than in the palaeo notch (Supplementary Table S1). The elevations of the palaeo tidal notch were measured at the base of the notch, and the respective *Wf* was added to calculate the palaeo RSL (Fig. 2a,b).

For the age attribution of the measured palaeo notches to MIS 5e, we consider that the corals on the fossil reef platform immediately below the notch (Fig. 2a) lived at the time, when the notch was cut into older Pleistocene limestones. This is similar to what happens today, with corals living a few meters below sea level (below 3–4 m depth, Fig. 2a,f) as the modern tidal notch is carved into older coral limestone (Fig. 2a,c–f). At site 4 (Fig. 1b), we sampled a fossil *Montastraea* sp. coral (BON-39-A; 12.2104°N, 68.3212°W) at + 2.65 ± 0.36 m (Fig. 2a,d). Two subsamples of this coral yielded U-series ages of 139.8 ± 4.5 ka and 147.3 ± 3.6 ka (Supplementary Table S4). According to the screening criteria applied in Obert *et al*.^38^ for ^230^Th/U-dating of other MIS 5e corals from Bonaire, the initial ^234^U/^238^U activity ratios of both subsamples of the fossil *Montastraea* sp. coral are higher than expected from the modern seawater value. In addition, the U content of both subsamples is relatively low, probably indicating post-depositional U loss.

### Modern and palaeo tidal simulations

**Figure 3:**
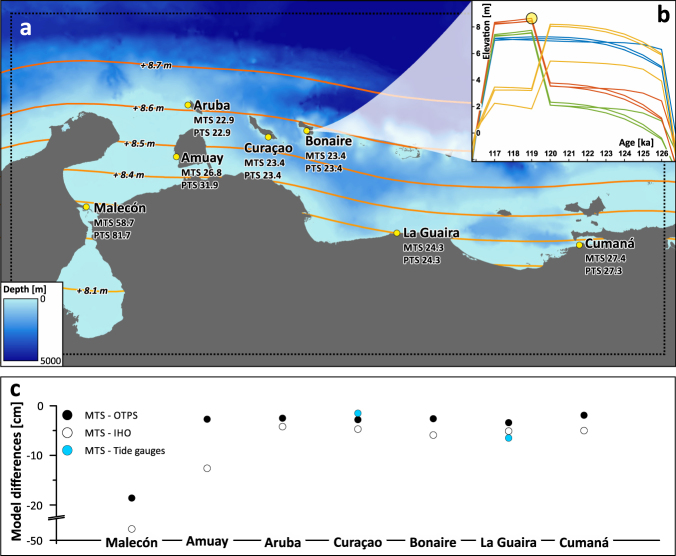
Results of modern and palaeo tidal models. (**a**) Boundary of modelled area (dotted line) and GEBCO_2014 bathymetry used in the MTS simulation. The yellow points indicate the sites where tidal predictions were extracted, with indication of the modelled Great Diurnal Range (in cm) from the modern (MTS) and the palaeo tidal simulations (PTS). Coloured contours represent the maximum palaeo RSL predicted by the GIA model for 119 ka (corresponding to the circle in **b**). (**b**) Relative sea-level curves for Bonaire as predicted from the ANICE-SELEN GIA model^41^ with the different mantle viscosity profiles and the four ESL scenarios described in Lorscheid *et al*.^2^. We chose the highest predicted sea level from this set of models (circle at ca. 119 ka) and added the gridded RSL prediction to the initial bathymetry. (**c**) Differences in the Great Diurnal Range between the model results and comparison datasets for all locations shown in (**a**). Comparison data from tide gauges are only available for Curaçao and La Guaira (The map has been created with the software ESRI ArcMap 10.4.1 [http://www.arcgis.com] using data from the GEBCO_2014 grid for background bathymetry [http://www.gebco.net/]).

To calculate the modern and palaeo tidal range (GT), we ran two tidal simulations (see Methods). The only difference between these simulations is the input topography. The Modern Tide Simulation (hereafter MTS) uses the GEBCO_2014 topography^40^. The Palaeo Tide Simulation (hereafter PTS) uses a palaeo terrain model calculated adding the maximum relative sea level predicted by the ANICE-SELEN GIA model^41^ for the southern Caribbean Region (Fig. 3a,b) to the GEBCO_2014 topography.

The GT at most locations show only minor differences (0.1 cm or less) between the MTS and PTS calculations (Fig. 3c, Supplementary Table S3). The only exceptions are the Malecón and Amuay stations (both located close to the very large shelf of the Gulf of Venezuela, Fig. 3a). At these stations, the GT calculated by the PTS is 23.6 and 4.2 cm higher, respectively, than the tidal range calculated by the MTS. This means that changes in palaeo tide at Malecón and Amuay are in the range of 15–28% with respect to the modern tidal range. In Kralendijk (Bonaire), located five kilometres south-east of the surveyed notches, the MTS and PTS both predict a GT of 23.4 cm.

## Discussion

### Dating and elevation of tidal notches

The results of U-series dating suggest that both coral subsamples taken from the deposit below the notches are diagenetically altered and biased towards older ages. Thus, they need to be regarded cautiously. Nevertheless, the coral ages can be correlated with those found along the north-eastern and eastern coast of Bonaire, that are constrained to MIS 5e through strictly reliable ^230^Th/U-ages of seven *Diploria strigosa* coral colonies collected at elevations between ~1.5 and ~5.5 m^37–39^. The stratigraphy of the Lower Terrace is very similar between the western coast (investigated in this study) and the north-eastern and eastern coasts, the only difference being the width of the fossil reef terrace, which is much larger on the eastern and northern, windward side of the island. Thus, it is reasonable to assume that the measured palaeo tidal notch was formed in MIS 5e, but it is not possible to pinpoint a specific time within MIS 5e (i.e. early or late in the interglacial) from our samples. However, it is most likely that the reef formed at peak MIS 5e sea level, as no higher reef or deposits correlated with MIS 5e can be found in this area. According to the GIA models employed here (see Methods), the MIS 5e RSL reached its peak on Bonaire at 119 ka (Fig. 3b).

The elevation of the deepest point of the notch, which represents the palaeo RSL elevation^3^, varies between +6.79 ± 0.18 m in the south-east and +7.37 ± 0.12 m in the north-west (Supplementary Table S1). Applying a linear fit, we calculate a relative tilting of 192 mm/km to the south-east. This is <1° of tilting and therefore at odds to the 20–30° regional tilting reported by Hippolyte and Mann (2011)^31^ for the Leeward Antilles as the result of long-term Pliocene to Quaternary compression on the island.

### Notch geometry

As briefly summarized in the introduction, the width of a tidal notch is correlated with the GT. In general, empirical evidence shows that the notch is always wider than the GT at the location where the notch is carved^14^. This relationship is maintained also on Bonaire, where our MTS calculated a GT of 23.4 cm (consistent with other independent datasets and models, see next section) and the modern notch is 81 ± 13 cm wide. On average, the palaeo tidal notch is slightly narrower than the modern one (65 ± 19 cm), but within error, the modern and palaeo tidal notch have a very similar width, suggesting that tidal range in MIS 5e was similar to today. This is supported by the results of our palaeo model simulation, which predicts a palaeo GT in Bonaire equal to the modern one.

The large variance that we measured between the width of palaeo and modern notches in Bonaire may reflect intra-site variability related, for example, to limestone dissolution processes at the palaeo notch (as observed at many locations in Bonaire^32^). Also, the biological rim that is present in the modern notch, and was most likely eroded with time in MIS 5e notches, might affect the discrepancy in width that we measured between modern and palaeo notches. As shown in Antonioli *et al*.^14^ also differences in lithology may cause the width of the notch to change, but the MIS 5e limestone, in which the modern notch is carved into, and the older Pleistocene limestone, in which the MIS 5e notch is carved into, are very similar in consistence. In addition, the larger width of the modern notch could be a result of a longer exposure to sea level at this elevation, as suggested by the slightly higher notch depth in the modern notch.

### Tidal modelling

The basic assumption behind our palaeo tidal model is that the tidal constituents during MIS 5e are equal to today. This is in line with the fact that tidal range changes during the last glacial cycles are mostly related to the different geometric settings of coastal areas under different sea levels^20^. Changes in tidal constituents driven by the gravitational influence of the moon and sun, are considered of importance only on much longer timescales (Miocene and older)^42^.

The geometry of the notches measured on Bonaire supports the model result that indicates virtually no changes between modern and palaeo GT at this location (Fig. 2g,h). As our tidal model is forced by satellite altimetry data and a very coarse bathymetry, we explore the uncertainties in predicting the modern GT through comparison with other independent datasets (Fig. 3c). Within the modelled area, the only available tide gauge data are from Curaçao and La Guaira. Although data at these stations do not span an entire tidal cycle, our model compares well with both stations (blue circles in Fig. 3c), and differences between modelled and observed tides are −1.5 and −6.4 cm, respectively. A second-order comparison can be done against the GT as calculated from water level timeseries derived from tidal constituents available from the International Hydrographic Organization (IHO^43^) and from the OSU Tidal Prediction Software (OTPS^29^). The differences between our modelled GT and that obtained from these two sources (respectively white and black circles in Fig. 3c) are generally less than 6 cm, with our model always underestimating the GT. The only exception is represented by the Malecón and Amuay stations, for which some comparisons show departures from our modelled values up to 48 cm (in Malecón, Fig. 3c).

**Figure 4:**
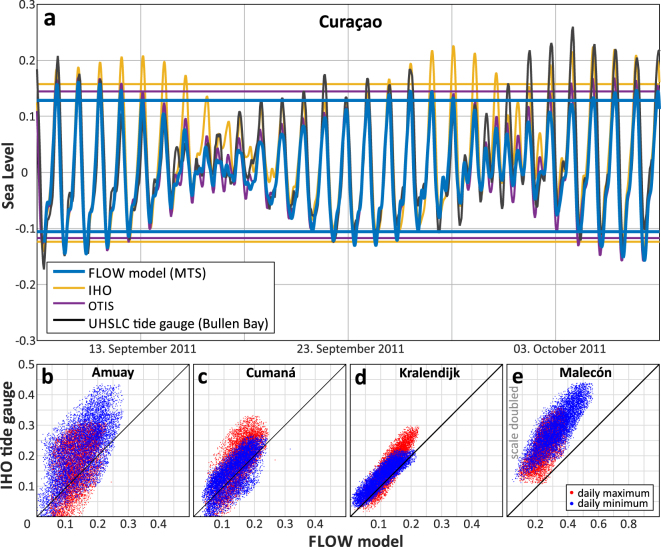
Comparison of modern tidal simulation and independent tidal datasets. (**a**) Tidal graph for Curaçao in September 2011. The graph shows the water level and the values for MHHW and MLLW for the MTS output as well as for the comparison datasets (tide gauge data is not referred to MSL). (**b**–**e**) Correlation graph for the daily maxima and minima between the MTS output and the observational IHO data for the locations (**b**) Amuay, (**c**) Cumaná, (**d**) Kralendijk and (**e**) Malecón (notice different scales).

The comparison between daily water level extremes (Fig. 4b–e) confirms that the MTS fits the water levels calculated using the IHO tidal constituents generally well, with the exception of the Malecón site (Fig. 4e). In addition, at the Amuay site there is some scatter between MTS and IHO water levels. The same pattern is observed when we compare our MTS results with the OTPS dataset. In both comparisons the daily minima are better represented than the daily maxima. Besides the extreme values, the diurnal shape of the tidal curves also shows a good correlation (Fig. 4a). The same typical diurnal shape is represented in the MTS results, the IHO and OTPS datasets and the tide gauge measurements, although the amplitudes vary.

In summary, our modern tidal model underestimates the modern tidal range by 5–20% on the Leeward Antilles and along the open Venezuelan coastline. In the very shallow Gulf of Venezuela (less than 50 m depth for a distance of up to 100 km), the difference between GT derived from tidal constituents and our MTS raises up to 45% (Fig. 3c). In these areas, the larger discrepancy is probably due to the coarse bathymetry we used in our model: as already recognized by other studies addressing tidal range changes^23^. Better predictions may be achieved using a higher-resolution bathymetry and a finer model grid size than the ones used here.

### MIS 5e sea levels and palaeotides

The geometry of notches measured on Bonaire and our tidal models highlight some important points concerning the study of past sea levels and the importance of changes in palaeo tides in the Last Interglacial:

- For Bonaire, field data and models show that changes in palaeo tidal range are negligible, while at sites located along wide and shallow continental shelves, such as Amuay and Malecón, changes in tidal range since MIS 5e might be instead relevant. At these two sites, our models are less accurate, but they show that the palaeo tidal range was 15–28% greater than modern one, as a result of the different bathymetry calculated by GIA models.
- Studies on Holocene RSL indicators show that changes in palaeo tidal range might affect the quantitative estimate of the indicative meaning, and hence the reconstruction of palaeo RSL^23^. In MIS 5e, this potential uncertainty has been rarely, if ever, considered. Our results show that changes in the tidal range in MIS 5e might instead be significant, depending on the broader geographic setting of the study area considered. In absence of information or estimates of the palaeo tidal range, we therefore suggest that MIS 5e RSL indicators that have their indicative meaning tied to tidal datums (i.e. cheniers, coral reef terraces, lagoonal deposits, shore platforms and tidal notches)^3^ must evaluate the possibility that tidal range changed since MIS 5e.
- There are two viable approaches that can be used when trying to estimate MIS 5e palaeo tides. If modern and palaeo tidal notches are available at the same location within the area of interest, measuring their geometric properties as described here might give a first-order estimate of GT changes in the palaeo record. If no tidal notches are preserved at the location of interest, a quantitative estimate of palaeo tidal range change can be given using a simple model such as the one described here, forced with global datasets and the best available bathymetry updated using GIA model outputs. This latter approach is not devoid of uncertainties that should be always evaluated comparing a model run simulating modern tidal ranges and comparing them with the best available tidal datasets.
- If it is not possible to use one of the two methods suggested here to evaluate palaeo tidal range changes, we suggest to consider an additional uncertainty on the indicative meaning of at least up to 15% of the modern tidal range (equal to the Amuay tidal range change shown in Fig. 3c). This value might be increased up to 30% in areas characterized by a large and shallow continental shelf.

## Methods

### Tidal notch geometry

We surveyed the geometry of the modern and a palaeo tidal notch at six sites along the south-western coast of Bonaire (Fig. 1b) at a regular distance of ca. 300 m. At each site, we measured the geometry of three notch profiles located a few meters from each other for both the modern and the palaeo tidal notch, resulting in a total of 36 tidal notch profiles. Notch measures were undertaken with a metered rod. Recorded values describe the vertical and horizontal distance from the deepest eroded point of the notch to the foremost point of the notch roof (**Wr** and **Dr** in Fig. 2b) and to the foremost point of the notch floor (**Wf** and **Df** in Fig. 2b). For the geometric elements of the notch, we here adopt the definitions of Antonioli *et al*.^14^. We define **Dr** as the *depth* of the tidal notch, while the distance **Wr + Wf** is defined as the notch *width*. For the fossil tidal notches, the elevation of the notch floor was measured once per location with a differential GPS system receiving OmniSTAR G2 real-time corrections, and is presented above the EGM08 geoid. To avoid bad GPS signal reception in proximity of the cliff, we installed the GPS antenna on a tripod on the platform before the cliff and used a laser pointer to measure the remaining vertical difference to the notch floor (Fig. 2a).

### ^230^Th/U-dating

In order to determine the age of the notches, a 4 × 4 × 3 cm piece of a coral skeleton was separated with a hammer from a fossil *Montastraea* sp. colony (BON-39-A) located on the platform directly below the palaeo notch (+2.65 ± 0.36 m, 12.210419°N, 68.321235°W) at site 4 (Fig. 1b). This *Montastraea* sp. coral forms an integral, cemented part of the Lower Terrace and, consequently, has been likely sampled *in situ*. Subsamples for dating were obtained from the most well-preserved skeletal parts of the coral in the laboratory using a diamond-coated micro-cutting disc. The average sample mass was ca. 0.15 g. After brief leaching in weak HNO_3_ in order to remove surface contamination, chemical separation of U and Th isotopes was performed as described by Yang *et al*.^44^. Uranium and Th isotopes were analysed using a MC-ICP-MS (Nu Plasma) at the Max Planck Institute for Chemistry, Mainz. Analytical details are described by Obert *et al*.^38^. Details about the calibration of the mixed U-Th spike are given by Gibert *et al*.^45^. To account for the potential effects of detrital contamination, all ages were corrected assuming an average upper continental crust ^232^Th/^238^U weight ratio of 3.8 for the detritus and ^230^Th, ^234^U and ^238^U in secular equilibrium. All activity ratios were calculated using the half-lives from Cheng *et al*.^46^ and all ages are reported at the 2σ-level.

### Tidal Modelling

To model the Great Diurnal Range in the wider Leeward Antilles-Venezuela region (Fig. 3a), we used the software Delft3D-FLOW. The simulation setup was done using the software Delft Dashboard v2.01 (Supplementary Table S2). The inputs of the model are a bathymetric-topographic raster and the astronomical forcing. The model extent is around 1080 × 560 km large and has a grid size of 0.03° (ca. 3.2 km). This area stretches from the Guajira Peninsula in the West to the Isla Margarita in the East and from the Venezuelan coastline to the abyssal plain of the Venezuelan Basin (Fig. 3a).

We ran two different simulations, the first using the present-day bathymetry and the second using a palaeo bathymetry. As modern bathymetry and topography we used the GEBCO_2014 dataset^40^ with a resolution of ca. 1 km. As palaeo bathymetry we used the GEBCO_2014 modified with results from the ANICE-SELEN coupled ice-earth model^41^, representing the glacial isostatic adjustment (GIA) at 119 ka, which is the highest point sea level reached in our configurations (Fig. 3b). The GIA simulation uses an eustatic sea-level rise of 2.5 m from the Greenland ice-sheets early and an additional of 5.5 m from the Antarctic ice sheet later in the interglacial. The mantle is divided into three zones with different viscosities between 0.5 × 10^21^ and 5.0 × 10^21^ Pa s (see melting scenario 3 and mantle viscosity 2 in Lorscheid *et al*.^2^). This simulation only shows one example of the possible range of ice melting scenarios and mantle viscosities that can be used for modelling the isostatic respond during this interglacial.

As astronomical boundary conditions for both simulations we used the global tidal inverse solutions TPXO7.2^29,30^. Both simulations were performed with a 5 minutes interval over 19 years (1998–2017), in order to include a full tidal cycle^47^. Monitoring stations were set at locations, where tide gauge data from stations of the International Hydrographic Organization (IHO) were available.

We used these locations to evaluate the present-day bathymetry simulation against data from IHO tide gauge stations and the OSU Tidal Prediction Software (OTPS^30^). Furthermore, we compared our results to observational data (Fig. 3c) from two tide gauges in Bullen Bay, Curaçao (January 2011 to April 2012), and La Guaira, Venezuela (January 1985 to December 1994), both maintained by the University of Hawaii Sea-Level Center (UHSLC, data from http://uhslc.soest.hawaii.edu/data/?rq#ned). The data for the IHO tide stations for the entire modelled timeframe was extracted directly from Delft Dashboard. As input for the OTPS we used the global TPXO8-atlas data (downloadable on the website: http://volkov.oce.orst.edu/tides/tpxo8_atlas.html) and predicted the tidal curves between 1998 and 2017 for each of the observational stations.

The tidal datums of MHHW and MLLW were calculated by averaging the daily maximum and minimum values through a 19 years cycle (1998–2017) to consider changes in the lunar cycle of 18.6 years^47,48^.

## Electronic supplementary material

Supplementary Tables S1–4
